# Baseline HCV Antibody Prevalence and Risk Factors among Drug Users in China’s National Methadone Maintenance Treatment Program

**DOI:** 10.1371/journal.pone.0147922

**Published:** 2016-02-23

**Authors:** Changhe Wang, Cynthia X. Shi, Keming Rou, Yan Zhao, Xiaobin Cao, Wei Luo, Enwu Liu, Zunyou Wu

**Affiliations:** National Center for AIDS/STD Control and Prevention, Chinese Center for Disease Control and Prevention, Beijing, China; Temple University School of Medicine, UNITED STATES

## Abstract

**Background:**

Hepatitis C virus (HCV) is the most common viral infection among injecting drug users worldwide. We aimed to assess HCV antibody prevalence and associated risk factors among clients in the Chinese national methadone maintenance treatment (MMT) program.

**Methods:**

Data from 296,209 clients who enrolled in the national MMT program between March 2004 and December 2012 were analyzed to assess HCV antibody prevalence, associated risk factors, and geographical distribution.

**Results:**

Anti-HCV screening was positive for 54.6% of clients upon MMT entry between 2004 and 2012. HCV antibody prevalence at entry declined from 66.8% in 2005 to 45.9% in 2012. The most significant predictors of HCV seropositivity were injecting drug use (adjusted odds ratio [AOR]: 8.34, 95% confidence interval [CI]: 8.17–8.52, p<0.0001) and a history of drug use ≥9 years (AOR: 2.01, 95% CI: 1.96–2.06, p<0.0001). Being female, of Uyghur or Zhuang ethnicity, and unmarried were identified as demographic risk factors (all p-values<0.0001). Of the 28 provincial-level divisions included in the study, we found that 5 divisions had HCV antibody prevalence above 70% and 20 divisions above 50%. The HCV screening rate within 6 months after MMT entry greatly increased from 30.4% in 2004 to 93.1% in 2012.

**Conclusions:**

The current HCV antibody prevalence remains alarmingly high among MMT clients throughout most provincial-level divisions in China, particularly among injecting drug users and females. A comprehensive prevention strategy is needed to control the HCV epidemic among MMT clients in China.

## Introduction

Hepatitis C virus (HCV) is a leading cause of chronic liver disease, including hepatitis, cirrhosis, and hepatocellular carcinoma [1–4]. Estimates of the HCV antibody prevalence among the Chinese general population have varied from 0.43% to 2.2%, corresponding to a range of 6 million to 30 million people [5–7]. While HCV transmission can occur via a variety of modes, the most common route is injecting drug use [3], and HCV is the most common viral infection among injecting drug users (IDU) worldwide[8, 9]. China’s drug user (DU) population has been estimated to be as high as 3.5 million, most of whom are opioid users and use drugs by injecting [10]. Thus, it is not surprising that HCV antibody prevalence among DU in China is thought to be very high, ranging from 15.6% to 98.7% in different geographical areas [11]. Meta-analyses have estimated a national HCV antibody prevalence of 67.0% among IDU and 60.1% among DU in methadone maintenance treatment (MMT) [12, 13].

Although implemented in response to the rapidly growing HIV/AIDS epidemic, China’s National MMT Program [14] also screens clients for anti-HCV upon enrollment and refers those identified as HCV seropositive to appropriate healthcare facilities. The MMT program started with 8 pilot sites in 2004 and has since rapidly expanded to become the world’s largest opioid-substitution treatment network [15]. By the end of 2012, there were 756 MMT clinics in China that had served about 380,000 clients cumulatively [16]. The numbers of MMT clinics vary from 1 to 71 among provincial-level divisions (defined as provinces, autonomous regions, and municipalities). Counties or districts where there are over 500 registered opioid users must have at least one established MMT clinic.

The national HCV antibody prevalence among Chinese MMT clients has not been previously reported. Given the high transmissibility of HCV among IDU, the overall HCV antibody prevalence among MMT clients is thought to be consistently high nationwide, particularly in provincial-level subdivisions with high rates of injecting drug use. We aim to assess the HCV antibody prevalence, risk factors, and geographical distribution among China’s MMT client population. The secondary aim is to assess the proportion of MMT clients who have accessed HCV antibody testing.

## Materials and Methods

### Study design and participants

The study cohort comprised all drug users enrolling in the national MMT program between March 2004 and December 2012. Participants were excluded if they could not be tracked by a unique client identification number (e.g., if the same identification number was incorrectly assigned to multiple clients or if the number contained errors). For clients who had dropped out of MMT and re-entered at a subsequent date, we restricted our analysis to the earliest enrollment record. Current guidelines state that all new clients should be screened for HCV antibody upon enrollment and that HCV-negative clients should be tested once every 12 months. Antibody-based testing is provided to all clients free of charge.

### Data collection

Data from this study were abstracted from the national MMT data system, which is one of eight subsystems of the national unified data collection system, the China National HIV/AIDS Comprehensive Response Information Management System (CRIMS). CRIMS is managed by the National Center for AIDS/STD Control and Prevention (NCAIDS) of the Chinese Center for Disease Control and Prevention (Chinese CDC) and has been described in detail elsewhere [17]. In brief, the MMT data system is updated daily with service records from each clinic. Local clinics complete baseline assessments at the initial visit to collect information on demographics, medical history, and drug use behavior. From this database, the following data were collected for clients >16 years of age for this study: sex, date of birth, ethnicity, education level, marital status, occupation, years of drug use, and injecting and needle sharing history, HIV serostatus, HCV serostatus and test dates.

### Statistical Analyses

We tabulated estimates of provincial- and national-level HCV antibody prevalence. The primary data analyses were limited to clients >16 years old who had a HCV test result at baseline, which we defined as within 6 months after entry. A binomial distribution, which was normally approximated, was assumed for 95% confidence intervals (CI) for prevalence estimates. Unadjusted odds ratios (OR) were generated by a logistic regression model for each covariate. A test for trend was used to assess the association between years of past drug use and HCV antibody prevalence. Adjusted odds ratios (AOR) were estimated from a multivariable logistic regression model. Variables with p<0.01 under univariate regression analysis were included in the multivariable model as potential confounders. All statistical analyses were performed using SAS software (Version, 9.3, SAS Institute Inc.,USA). All reported CIs are 95%, and p-values are two-sided.

### Ethical approval

This study was reviewed and approved by the Institutional Review Board of NCAIDS. As all data included here were collected during the regular administration of China’s National MMT Program, and all participants in this program provided informed consent at the time of enrollment, no further study-specific informed consent was sought. Data from participants younger than 16 were only used to calculate provincial- and national-level HCV screening coverage and HCV antibody prevalence and were otherwise excluded.

## Results

### Study Cohort

**Fig 1:**
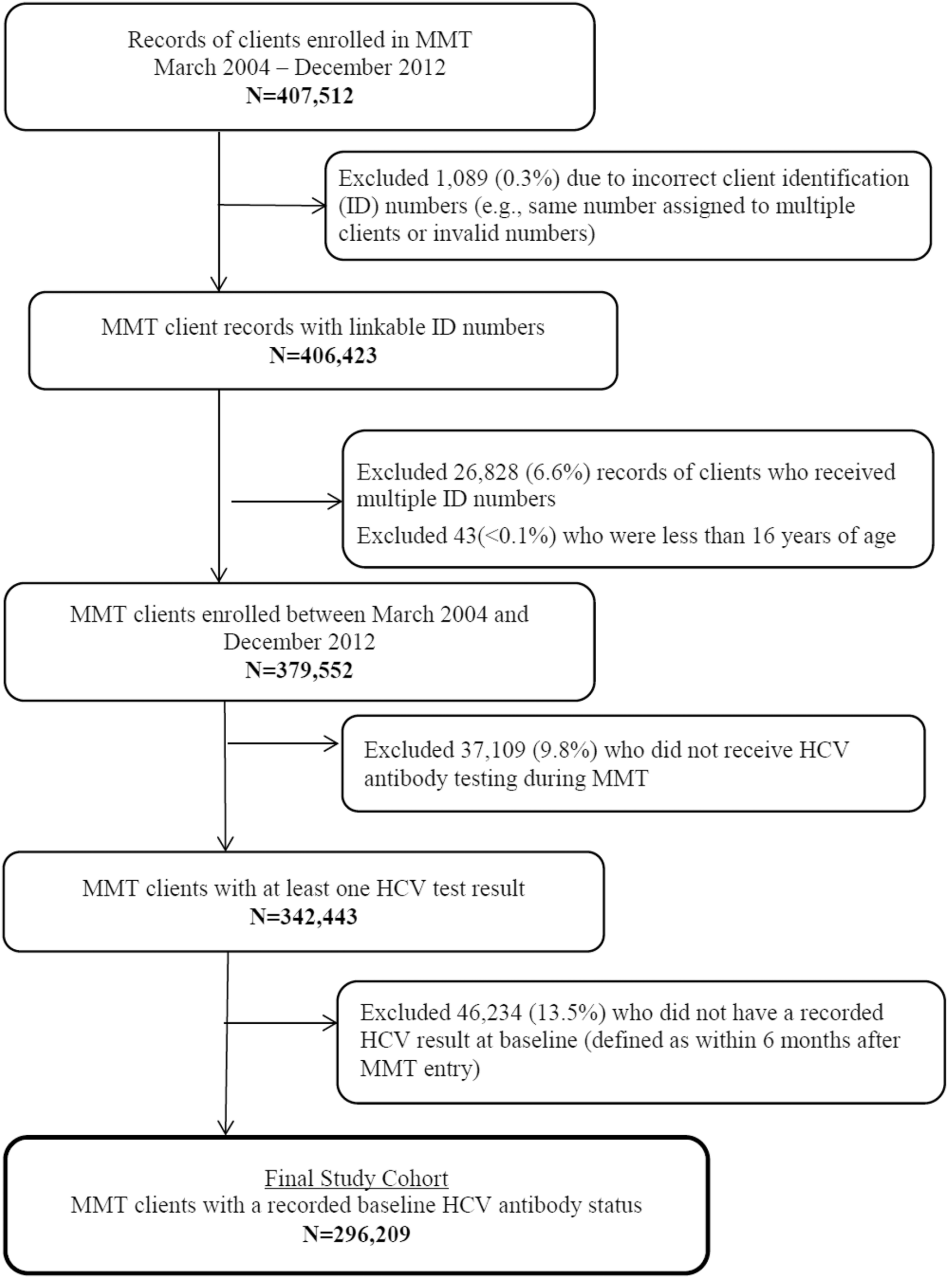
Development of the study cohort.

The development of the study cohort is presented in Fig 1. Between March 2004 and December 2012, 379,552 drug users had been enrolled into MMT, and 342,443 MMT clients (90.2%) had received a HCV antibody test at least once. Among these clients, 46,234 (13.5%) were excluded from the primary study analyses because their first HCV test result did not occur within 6 months after entry. The final study cohort was composed of 296,209 participants.

#### HCV antibody prevalence at baseline

In the initial year of the MMT program in 2004, only 1,438 clients were enrolled. The number of clients enrolled quadrupled in the second year and again in the third year. The peak of newly enrolled clients was 76,528 individuals in 2008, which then decreased from 66,871 in 2009 and to 40,596 in 2012 (Table 1). The proportion of self-reported IDU was stable at 81% in the first three years (2004–2006), but decreased to 76.1% in 2007 and reached a low of 56.0% in 2012. The proportion of clients who obtained HCV testing was less than one-third in 2004 (30.4%) and 2005 (27.8%) and then increased from 59.1% in 2006 and to 93.1% in 2012. The HCV antibody prevalence among overall clients enrolled in 2004 was 34.6%. The HCV antibody prevalence sharply increased to 66.8% in the second year of 2005 but has shown steady annual declines up to the last year of the study in 2012 (45.9%). The HCV antibody prevalence among self-reported IDU peaked at 74.1% in the second year and was stable from 2006–2011 at 70–74% before falling slightly to 68.9% in 2012.

**Table 1:** Self-reported injecting drug use (IDU), HCV antibody testing, and HCV antibody prevalence among newly enrolled MMT clients by year, 2004–2012.

	2004	2005	2006	2007	2008	2009	2010	2011	2012	Total
Clients newly enrolled into MMT	1438	5440	28747	58783	76528	66871	52521	48628	40596	379552
Self-reported IDU (%)	81.2	81.1	81.6	76.1	72.3	66.6	60.6	59.3	56.0	67.7
HCV testing within 6 months after enrollment (%)	30.4	27.8	59.1	68.7	75.8	79.4	83.6	90.7	93.1	78.0
Baseline HCV antibody prevalence % (95% CI)	34.6 (30.1–39.0)	66.8 (64.4–69.2)	64.8 (64.1–65.5)	61.7 (61.2–62.2)	59.5 (59.1–59.9)	54.9 (54.5–55.3)	49.9 (49.4–50.3)	49.2 (48.8–49.7)	45.9 (45.4–46.4)	54.6 (54.4–54.8)
HCV antibody prevalence among clients self-reporting IDU* % (95% CI)	37.4 (32.6–42.3)	74.1 (71.7–76.5)	72.6 (71.9–73.4)	72.9 (72.4–73.4)	73.2 (72.8–73.6)	71.9 (71.4–72.4)	70.5 (69.9–71.0)	70.9 (70.3–71.4)	68.9 (68.3–69.5)	71.7 (71.5–71.9)

CI: confidence interval

*Among clients with baseline HCV antibody test results.

### Geographical Distribution

The utilization of MMT and the HCV antibody prevalence varied significantly by provincial-level division (Table 2). Five provincial-level divisions had more than 30,000 MMT clients, and three had less than 1,000. Eight divisions had more than 10,000 HCV antibody-positive MMT clients.

**Table 2:** Self-reported injecting drug use (IDU), HCV antibody testing, and HCV antibody prevalence among newly enrolled MMT clients by provincial-level division, 2004–2012.

Division	Number of MMT clients	Self-reported IDU (%)	Received anti-HCV test at least once (%)^†^	HCV seropositivity (%)^‡^
Anhui	3494	2603 (74.5)	3281 (93.9)	1746 (56.6)
Beijing	3264	2578 (79.0)	3065 (93.9)	1695 (63.4)
Chongqing	21894	18940 (86.5)	21016 (96.0)	14166 (74.5)
Fujian	8179	5670 (69.3)	7657 (93.6)	3773 (52.4)
Gansu	16178	3122 (19.3)	15433 (95.4)	2176 (15.3)
Guangdong	28656	23839 (83.2)	25705 (89.7)	16535 (69.5)
Guangxi	31013	25405 (81.9)	29060 (93.7)	18174 (68.9)
Guizhou	37514	18703 (49.9)	32846 (87.5)	12257 (45.7)
Hainan	13557	8754 (70.3)	11175 (82.4)	3883 (40.4)
Hebei	378	343 (90.7)	369 (97.6)	269 (73.9)
Heilongjiang*	-	-	-	-
Henan	6209	891 (14.4)	5888 (94.8)	403 (7.2)
Hubei	21725	17493 (80.6)	19835 (91.3)	11588 (68.3)
Hunan	31205	24109 (77.3)	27897 (89.4)	15140 (60.2)
Inner Mongolia	4146	1934 (46.6)	3954 (95.4)	1241 (32.5)
Jiangsu	8764	7440 (84.9)	8546 (97.5)	5851 (71.7)
Jiangxi	5119	4458 (87.1)	4559 (89.1)	2692 (69.2)
Jilin	323	306 (94.7)	275 (85.1)	141 (65.0)
Liaoning*	-	-	-	-
Ningxia	5012	1822 (36.4)	4543 (90.6)	1038 (26.4)
Qinghai	1266	846 (66.8)	1225 (96.8)	783 (67.5)
Shandong	67	59 (88.1)	36 (53.7)	21 (72.4)
Shanghai	6188	4722 (76.3)	5365 (86.7)	2966 (66.4)
Shannxi	21171	13140 (62.1)	19391 (91.6)	8555 (50.0)
Shanxi	4583	365 (8.0)	4103 (89.5)	212 (5.6)
Sichuan	34839	23434 (67.3)	29610 (85.0)	11227 (50.2)
Tianjin	1411	1308 (92.7)	1389 (98.4)	1072 (78.1)
Tibet*	-	-	-	-
Xinjiang	13104	11181 (85.3)	11886 (90.7)	7019 (68.5)
Yunnan	35427	23663 (66.8)	31167 (88.0)	12541 (54.3)
Zhejiang	14866	7853 (52.8)	13168 (88.6)	4523 (38.7)
**Total**	**379595**	**254981 (67.7)**	**342443 (90.2)**	**161687 (54.6)**

*Division did not have an MMT clinic by the end of 2012.

^†^ Based on MMT clients who received at least one HCV antibody test (baseline or follow-up test).

^‡^Based on MMT clients with baseline HCV antibody test results.

**Fig 2:**
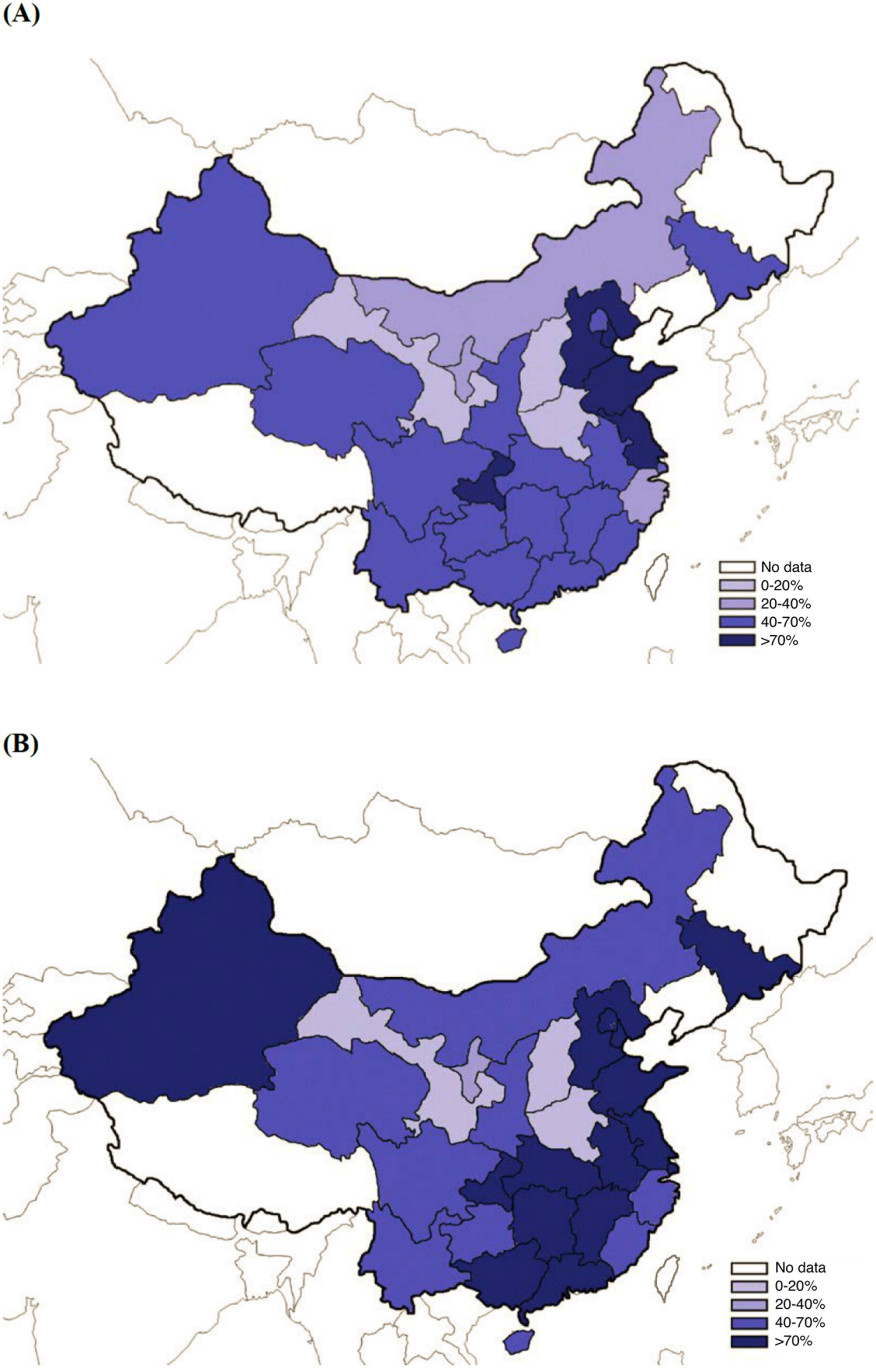
The geographical distribution of (A) HCV antibody prevalence and (B) proportion of injecting drug use among MMT clients with baseline HCV test results in China.

The baseline HCV antibody prevalence ranged from a low of 5.6% in Shanxi province to a high of 78.1% in Tianjin municipality. The distributions of HCV antibody prevalence and proportion of self-reported IDU nationwide are presented in Fig 2. Data were not available from some provincial-level divisions because the MMT program has not yet been established in those areas.

The demographic and drug use characteristics of the cohort are summarized in Table 3. The overall HCV antibody prevalence upon entry among the 296,209 MMT clients with baseline test results was 54.6% (95% CI: 54.5–54.8). Most clients were male (84.9%), middle-aged (30–44 years 64.6%), Han Chinese (85.7%), educated beyond middle school (79.2%), unmarried (52.3%), and unemployed (66.3%). The HIV antibody prevalence was 5.8%. More than half (51.1%) reported having used drugs for 9 or more years prior to enrollment in MMT. While two-thirds of clients self-reported drug use through injecting (66.8%), most reported that they had never shared needles (87.6%). HCV antibody prevalence was highest among HIV-positive clients (79.7%, 95% CI: 79.1–80.3) and those who had reported injecting drugs (71.7%, 95% CI: 71.5–71.9) or sharing needles (78.5%, 95% CI: 78.1–78.9).

**Table 3:** HCV antibody prevalence and risk factors among MMT clients with baseline HCV test results in China, 2004–2012.

	Participants, N (%)	HCV Cases, n	Prevalence, % (95% CI)	Unadjusted OR, (95% CI)	P-value	Adjusted OR, (95% CI)	P-value
***Demographic characteristics***							
**Gender**							
Male	251599 (84.9)	135395	53.8 (53.6–54.0)	1.0		1.0	
Female	44610 (15.1)	26292	58.9 (58.5–59.4)	1.23 (1.21–1.26)	<0.0001	1.39 (1.36–1.43)	<0.0001
**Age (years)**							
16–29	71881 (24.3)	35664	49.6 (49.2–50.0)	1.00		1.00	
30–44	191480 (64.6)	109550	57.2 (57.0–57.4)	1.36 (1.34–1.38)	<0.0001	1.35 (1.31–1.38)	<0.0001
≥45	32848 (11.1)	16473	50.1 (49.6–50.7)	1.02 (1.00–1.05)	<0.0001	1.28 (1.24–1.33)	<0.0001
**Ethnicity**							
Han	253947 (85.7)	140492	55.3 (55.1–55.5)	1.00		1.00	
Hui	7529 (2.5)	3089	41.0 (39.9–42.1)	0.56 (0.54–0.59)	<0.0001	0.77 (0.73–0.82)	<0.0001
Uyghur	7086 (2.4)	4900	69.1 (68.1–70.2)	1.81 (1.72–1.90)	<0.0001	1.34 (1.26–1.42)	<0.0001
Zhuang	8976 (3.0)	5435	60.6 (59.5–61.6)	1.24 (1.19–1.29)	<0.0001	1.29 (1.22–1.36)	<0.0001
Other	18631 (6.3)	7745	41.6 (40.9–42.3)	0.58 (0.56–0.59)	<0.0001	0.78 (0.75–0.81)	<0.0001
Missing data	40 (0)	26					
**Education level**							
≤ Primary school	61629 (20.8)	32446	52.6 (52.3–53.0)	1.00		1.00	
Middle school	170559 (57.6)	94927	55.7 (55.4–55.9)	1.13 (1.11–1.15)	<0.0001	1.02 (1.00–1.05)	<0.0001
≥ High school	63993 (21.6)	34294	53.6 (53.2–54.0)	1.04 (1.02–1.06)	0.0146	0.95 (0.92–0.98)	<0.0001
Missing data	28 (0)	8					
**Marital Status**							
Married	140732 (47.5)	67830	48.2 (47.9–48.5)	1.00		1.00	
Unmarried	155009 (52.3)	93600	60.4 (60.1–60.6)	1.64 (1.61–1.66)	<0.0001	1.25 (1.23–1.28)	<0.0001
Missing data	468 (0.2)	257					
**Occupation**							
Employed	99927 (33.7)	47516	47.6 (47.2–47.9)	1.00		1.00	
Unemployed	196252 (66.3)	114150	58.2 (58.0–58.4)	1.53 (1.51–1.56)	<0.0001	1.12 (1.11–1.15)	<0.0001
Missing data	30 (0)	21					
**HIV serostatus**							
Negative	254170 (85.8)	133180	52.4 (52.2–52.6)	1.00		1.00	
Positive	17200 (5.8)	13700	79.7 (79.1–80.3)	3.56 (3.42–3.70)	<0.0001	1.74 (1.67–1.82)	<0.0001
Missing data	24839 (8.4)	14806					
***Drug use history characteristics***							
**Duration (years)**							
0–2	55874 (18.9)	18611	33.3 (32.9–33.7)	1.00		1.00	
3–5	44525 (15.0)	20812	46.7 (46.3–47.2)	1.76 (1.71–1.80)	<0.0001	1.31 (1.27–1.35)	<0.0001
6–8	43063 (14.5)	24801	57.6 (57.1–58.1)	2.72 (2.65–2.79)	<0.0001	1.67 (1.62–1.72)	<0.0001
≥ 9	151408 (51.1)	96823	63.9 (63.7–64.2)	3.55 (3.48–3.63)	<0.0001	2.01 (1.96–2.06)	<0.0001
Missing data	1339 (0.5)	640	47.8 (45.1–50.5)				
**Injecting drug use**							
No	96824 (32.7)	19333	20.0 (19.7–20.2)	1.00		1.00	
Yes	197832 (66.8)	141810	71.7 (71.5–71.9)	10.15 (9.96–10.34)	<0.0001	8.34 (8.17–8.52)	<0.0001
Missing data	1553 (0.5)	544					
**Ever shared needles**							
No	259459 (87.6)	132830	51.2 (51.0–51.4)	1.00		1.00	
Yes	36747 (12.4)	28854	78.5 (78.1–78.9)	3.49 (3.40–3.58)	<0.0001	1.52 (1.48–1.57)	<0.0001
Missing data	3 (0)	3					
Total	296209	161687	54.6 (54.5–54.8)				

CI: confidence interval, OR: odds ratio.

### Risk factors associated with HCV infection

Risk factors associated with being HCV antibody positive are shown in Table 3. The risk was higher for participants who were female (AOR: 1.39, 95% CI: 1.36–1.43, p<0.0001), over 30 years (30–44 years AOR: 1.35, 95% CI: 1.31–1.38, p<0.0001; age group ≥45 years AOR: 1.28, 95% CI: 1.24–1.33, p<0.0001), of Uyghur or Zhuang ethnicity (Uyghur AOR: 1.34, 95% CI: 1.29–1.42, p<0.0001; Zhuang AOR: 1.29, 95% CI: 1.22–1.36, p<0.0001), having attained a middle school education (AOR: 1.02, 95% CI: 1.00–1.05, p<0.0001 compared to primary school), unmarried (AOR: 1.25, 95% CI: 1.23–1.28, p<0.0001), unemployed (AOR: 1.12, 95% CI: 1.11–1.15, p<0.0001), HIV-positive (AOR: 1.74, 95% CI: 1.67–1.82, p<0.0001), had a longer history of drug use (≥9 years AOR: 2.01, 95% CI: 1.96–2.06; 6–8 years AOR: 1.67, 95% CI: 1.62–1.72; 3–5 years AOR: 1.31, 95% CI:1.27–1.35, all p<0.0001and trend test p<0.0001), had reported injecting drug use (AOR: 8.34, 95% CI: 8.17–8.52, p<0.0001) and had ever shared needles (AOR: 1.52, 95% CI: 1.48–1.57, p<0.0001).

## Discussion

China’s national MMT program was established as part of an overarching strategy to stem the HIV epidemic. The national MMT program provides regular anti-HCV testing, and the HCV antibody prevalence has not been previously described. Although the HIV epidemic is largely concentrated in six provincial-level divisions, HCV antibody prevalence among MMT clients was presumed to be high nationwide due to its high transmissibility, particularly through injecting drug use. By utilizing the National MMT Program database, we presented an overview of the prevalence, risk factors, and geographical distribution of anti-HCV seropositivity in the Chinese MMT client population from the program’s inception in 2004 through 2012.

The primary strength of this observational study is the large sample size and access to the National MMT program database, which covers nearly all geographical regions in China. The length of the study period covers the entire existence of the Chinese MMT program from 2004 to 2012, which allows for monitoring of HCV antibody prevalence trends among newly enrolled MMT clients. Due to the large sample size and high coverage of areas, the results of this study allow for a comprehensive assessment of HCV epidemiology among DUs in China.

### HCV antibody prevalence

From 2004 to 2012, the overall HCV antibody prevalence for all MMT clients at enrollment was 54.6%. This is lower than the prevalence estimates from recent meta-analyses of 67.0% among IDUs and 60.1% among DUs in MMT [12, 13]. Drug injecting was reported by 66.8% of clients, and 12.4% reported needle sharing. The HCV antibody prevalence among IDUs in MMT in our cohort was higher (71.7%) than previous estimates of 61.4% and 66.97%, although it is worth noting that their studies focused on IDUs regardless of MMT enrollment [18, 19].

The HCV antibody prevalence in the Chinese MMT program is similar to estimates reported in MMT clinics in other countries. Most studies of HCV antibody prevalence among MMT clients in other countries (including the United States, Switzerland, and Australia) were limited to individual cities or MMT clinics [20–24]. These studies showed variance in HCV antibody prevalence among MMT clients ranging from 58% to 87%. Direct comparisons between sites and studies are unreliable due to the differences in study cohort profiles, particularly related to the percentages of IDU and non-injecting DU.

In China, the HCV antibody prevalence of clients upon MMT entry has declined each year from 2005 (66.8%) to 2012 (45.9%). (An exception is the first year of the MMT program in 2004, which reported a rate of 34.6%; this may be due to the very low HCV antibody screening coverage and unreliable lab results during the initial pilot period of the MMT program). It may be that the decreasing annual HCV antibody prevalence results is driven by the decreasing proportions of IDUs among newly enrolled MMT clients. Concurrently, the HCV antibody testing coverage at baseline has greatly increased from 30.4% in 2004 to 93.1% in 2012, which indicates a remarkable improvement in MMT health services and linkage to anti-HCV testing.

### Risk Factors

The factors most strongly associated with HCV infection were related to drug use history, which has been addressed in depth in the literature. The prevalence of HCV increased with longer histories of drug use [3, 25–27]. HCV infection was also much more likely to be present among IDUs and HIV-positive clients [3, 8, 19, 26].

One factor that has not been addressed thoroughly is the difference in anti-HCV prevalence by sex. Women had a higher prevalence of 58.9% compared to a prevalence of 53.8% for men. Other studies on HCV antibody and DU in China in the English and Chinese research literature were not at a consensus on sex as a risk factor [11, 18, 26–29]. In the national general population, there is no significant difference in HCV antibody prevalence between sex [30]. However, another study also suggests that sexual transmission of HCV may be more efficient in females [24]. Future research should address sex as a risk factor for HCV antibody positivity.

### Geographical Distribution

Our findings indicate that the HCV antibody prevalence among MMT clients is alarmingly high throughout China. Of the 28 provincial-level divisions included in our study, 20 reported rates above 50%, including 5 divisions with prevalence above 70%. HCV antibody prevalence showed extremely wide variations between divisions. We believe that these differences can be attributed in part to the different distribution of IDU in regions. In the provincial-level divisions with the two lowest HCV antibody prevalence, Henan and Shanxi, we found that these two provinces had very low percentages of clients who reported having injected drugs (Henan: 14.0%; Shanxi: 8.1%) compared to the national percentage (66.8%). Tianjin, the provincial-level division with highest rate of HCV antibody prevalence at 78.1%, also had a remarkably high percentage of IDUs (92.7%). Regions with high proportions of IDUs should be focal regions for controlling the HCV epidemic. Of all provincial-level divisions that have implemented MMT, eight had more than 10,000 HCV antibody positive MMT clients.

### Comparison to the HIV Epidemic

The Chinese MMT program was originally implemented as a response to the emerging HIV epidemic[14]. Historically and presently, the HIV epidemic in China has been largely concentrated in 6 provincial-level divisions: Yunnan, Guangxi, Henan, Sichuan, Xinjiang and Guangdong [31]. With the exception of Henan, these divisions lie along major drug trafficking routes and are the top five are as for HCV and HIV co-infection. While MMT has contributed to slowing the spread of HIV, MMT without other harm reduction strategies has not been as effective for controlling the HCV epidemic [32]. This situation is not unique to China; studies from many countries have noted that HCV has a higher transmission probability than HIV [25, 33, 34]. One example is Hong Kong, where under a long-established MMT program with over 90% coverage, HIV antibody prevalence was 0.3% and HCV antibody prevalence was 85% [23].

### Recommendations

Because many IDUs first begin drug use through non-injecting methods [35], implementing outreach efforts to this population of non-injecting DUs can potentially prevent transition to drug injecting [33]. Data showed a 71.7% HCV antibody prevalence for IDUs compared to 20.0% for non-injecting DUs. This HCV antibody prevalence for non-injecting DUs is comparable to other studies reporting rates of 2.3% to 35.3% [19, 36], and the difference in prevalence between IDUs and non-injecting DUs is similar to results from other studies [19, 33]. In our data, the low proportion of IDU in Henan and Shanxi is strongly correlated with the remarkably low HCV antibody prevalence in these two provinces. We recommend further investigation on what distinguishes these two provinces in this regard from the rest of the country.

Emphasizing routine testing for HCV antibody is critically important for MMT clients because a positive serostatus has clinical implications. Low levels of HCV knowledge has been cited as a barrier to antibody testing and treatment in China and abroad [37, 38]. Clients who were positive for HCV antibody have been shown to need significantly higher dosages of methadone [24]. Researchers have recommended that it would be beneficial for anti-HCV positive clients to receive treatment of HCV infection at methadone clinics[39], echoing a similar suggestion regarding antiretroviral therapy for HIV. Cost is a significant barrier to clients because unlike ART, neither methadone nor HCV treatment is free in China. MMT services costs up to 10 RMB per day (~$1.60 USD).

Finally, we recommend further expansion of China’s needle and syringe exchange programs to supplement MMT. Multi-component harm reduction strategies that include MMT and needle and syringe exchange programs have shown effectiveness is reducing the risk of HCV seroconversion up to 75–80% [32, 40]. In 2011, China had 913 needle and syringe exchange sites, which distributed 12 million needles and syringes to over 60,000 IDUs [16]. However, it is estimated that only about 2% of IDUs in China have been able to access needle exchange programs so far [41].

### Limitations

While our study relied on HCV antibody tests to determine infection status, it is notable that spontaneous resolution or viral clearance occurs in 15–20% of individuals, which varies by race and sex [42, 43]. Our study is limited to describing HCV antibody prevalence, instead of clinical presentation of active HCV. We were also limited to describing the HCV antibody prevalence upon entry to MMT, which is not directly reflective of all MMT clients. We could only include MMT clients who had at least one anti-HCV test result within 6 months in the primary analysis; due to the high early attrition rate in MMT, many clients drop-out without receiving HCV antibody testing [44, 45]. We excluded 13.5% of cases due to a lack of HCV antibody testing within 6 months of entry. Most excluded participants had enrolled in MMT at early stages of the program when anti-HCV testing rates were comparatively low. Compared with MMT clients without baseline HCV test results, MMT clients with a known baseline serostatus have a slightly lower proportion of self-reported IDU and needle sharing, and have a longer history of drug use. Also, some clients may acquire HCV during the course of MMT due to ongoing drug use [46]. As these four factors increase the risk for HCV infection, the HCV antibody prevalence of all MMT clients may be slightly higher than the prevalence among MMT clients who were tested at entry. However, we believe that these differences would not have a large impact on the final results nor would it alter our conclusions. We also acknowledge that the accuracy and reliability of data is dependent on the performance of local staff from clinics located around the country, including in resource-limited areas. Due to the different brands of anti-HCV tests, this may also have caused slight variations between different MMT clinics. We attempted to reduce the role of confounding variables by using a multivariable analysis model but as in all observational studies, there still remains a residual risk of confounding. Finally, due to the extremely large number of cases included in the analysis, we acknowledge that a relatively slight difference between groups can create a statistically significant estimate.

Our study concludes that the HCV antibody prevalence among MMT clients has reached alarmingly high rates in many areas within China, which is reflective of the high transmissibility of HCV. Routine antibody screening, treatment options that address drug use, HCV infection, and potential HIV/HCV co-infection, and continued expansion of MMT and needle and syringe exchange programs are critical components of a comprehensive national control and prevention strategy.
